# Aortic Dissection Presenting as Septic Shock: A Case Report and Literature Review

**DOI:** 10.1155/2018/9706290

**Published:** 2018-02-14

**Authors:** Jing Feng, Rui Liu, Sai Ma, Changkui Cao, Wei Zhang, Yang Zhao, Shinan Nie

**Affiliations:** ^1^Department of Emergency Medicine, Jinling Hospital, Medical School of Nanjing University, Nanjing 210002, China; ^2^Department of Cardiology, Xijing Hospital, Fourth Military Medical University, Xi'an, Shaanxi 710032, China

## Abstract

Acute aortic dissection is a life-threatening clinical emergency, which mostly occurs in aged patients and presents with sharp chest pain. In this paper, we reported a case of acute aortic dissection, which induced septic shock in a young woman, without typical chest pain. The septic shock was possibly due to the bacterial translocation caused by aortic dissection-induced intestinal ischemia and intestinal epithelial barrier dysfunction. Our case appeared as the first case report of aortic dissection presenting as septic shock. This case is rare but can serve as a reminder that aortic dissection may be accompanied by septic shock, and this could result in a grave outcome.

## 1. Introduction

Aortic dissection is a life-threatening emergency with high mortality, caused by an intimal and medial tear in the aorta, with formation of a false lumen within the aortic media. The prognosis of aortic dissection is grave with more than 50% mortality, due mainly to late diagnosis and inadequate treatment [1]. Clinically, the most common feature of aortic dissection is abrupt onset of sharp back or chest pain (>96% of cases).

Septic shock, which is a frequent cause of death in the intensive care unit, is defined as severe sepsis combined with hypotension unresponsive to adequate fluid resuscitation [2]. Clinically, sepsis is usually caused by severe infection or trauma. To date, there is no report of a clinical case of septic shock induced by aortic dissection.

Herein, we described a young female aortic dissection patient who firstly presented with symptoms of septic shock without typical chest pain, which led to delayed diagnosis and treatment of aortic dissection.

## 2. Case Report

**Figure 1:**
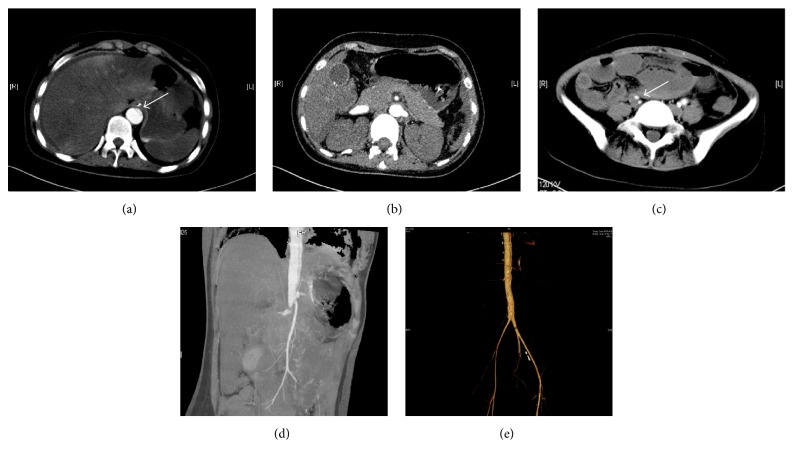
CTA images of aortic dissection. (a) CTA image shows the true and false lumen of aortic dissection and abnormal perfusion of liver and spleen. (b) Decreased perfusion of bilateral kidneys. (c) Abnormal right internal and external iliac arteries. (d and e) 3D reconstruction of CTA images.

A 23-year-old Chinese female was transferred to our emergency department for severe epigastric pain, accompanied by vomiting, diarrhea, gradual hypourocrinia, and right lower limb weakness. The patient had a past medical history of patent ductus arteriosus (PDA) transcatheter occlusion. Additionally, she had no past history of hypertension, Marfan syndrome, Ehlers-Danlos syndrome, or trauma. Laboratory data, including blood count and electrolytes, have shown leukocytosis and metabolic acidosis (shown in Table 1). The blood pressure was 80/55 mmHg (treated with norepinephrine). Electrocardiogram showed sinus tachycardia (132/min) and ST-T change. There was a reduction of muscle strength in the right lower limb, together with hypesthesia, ochrodermia, low skin temperature, and decreased dorsalis pedis artery pulse. Consequently, the diagnosis of septic shock and multiorgan failure was given. Therefore, treatment of fluid resuscitation and anti-infection therapy were applied immediately. However, her condition deteriorated, notwithstanding intensive treatment. In view of the past medical history of PDA, gradual hypourocrinia, and right lower limb weakness, we considered the possibility of aortic dissection. Computed tomography angiography (CTA) results (shown in Figure 1) confirmed our tentative diagnosis: thoracic and abdominal aortic dissection, thinning of bilateral renal arteries and right internal and external iliac arteries, and abnormal infusion of liver, spleen, and kidneys. Unfortunately, the patient's relatives declined treatment, since her condition had deteriorated by the time the diagnosis was confirmed. The patient died one day after being discharged from the hospital.

## 3. Discussion

In this paper, we reported a rare case of a young female with acute aortic dissection presenting as septic shock. Unfortunately, because of the young age and absence of typical symptoms, the diagnosis of acute aortic dissection was initially missed, leading to a delayed treatment and deteriorating multiorgan injury. This case appeared as the first report of aortic dissection presenting as septic shock.

Septic shock is defined as the presence of sepsis combined with refractory hypotension (systolic blood pressure of <90 mmHg, mean blood pressure of <90 mmHg, or systolic arterial pressure 40 mmHg below baseline), despite adequate fluid resuscitation [3]. Clinically, septic shock is commonly caused by severe infection or trauma [4]. In this case, however, no explicit infection sites were found, and symptoms in the right lower limb could not be explained by septic shock. Thus, the pathogeny of septic shock in this patient was further investigated.

Patients with aortic dissection typically present with sudden sharp tearing or stabbing chest pain, which was not present in this patient. Less common manifestations include shock and lower extremity ischemia [5]. Clinically, the diagnosis of aortic dissection is mainly based on the symptoms and confirmatory imaging evidence [6]. In this case, even though the symptoms of right limb ischemia and hypourocrinia were suggestive of the possibility of aortic dissection, this potential diagnosis was not considered initially, as this was a young female patient without significant medical history or any related risk factors (such as past history of hypertension, Marfan syndrome, Ehlers-Danlos syndrome, or trauma). Nevertheless, CTA was performed and the diagnosis of aortic dissection was eventually confirmed. Unfortunately, this diagnosis was missed initially, and the patient's relatives declined treatment due to her deteriorated condition.

In this case, septic shock was possibly due to bacterial translocation caused by aortic dissection-induced intestinal ischemia and intestinal epithelial barrier dysfunctions. Bacterial translocation is the passage of gastrointestinal bacteria to local lymph nodes or distant sites, which is associated with higher septic morbidity in patients [7]. As indicated by the CTA images, the area and pressure of the false lumen were larger than those of the true lumen, resulting in significant reduction of true lumen flow. This caused changes in the celiac, mesenteric, and renal artery, leading to the symptoms of severe epigastric pain, accompanied by vomiting, diarrhea, and gradual hypourocrinia, which resulted in intestinal epithelial barrier dysfunction and subsequent bacterial translocation-induced sepsis, and eventual multiorgan failure. Under normal conditions, intestinal epithelial barrier functions to control the passage and pathogenic components of luminal bacterium [8]. Upon injuries such as ischemia conditions, intestinal barrier defect could lead to the translocation of bacteria to the bowel wall and microcirculation [9]. In a recent clinical study raised by Gu et al., they demonstrated that intestinal barrier dysfunction played an essential role in systemic inflammatory responses in patients with aortic dissection by analyzing the inflammatory biomarkers of the serum samples [10]. Bacterial translocation has been reported under numerous conditions in animal models, but only a few papers have described bacterial translocation as a direct cause of septic shock in humans. In a case report by Tani et al., two patients developed septic shock directly caused by bacterial translocation [11]. Herein, our case suggested that aortic dissection could be a responsible cause for intestinal barrier defect, bacterial translocation, and subsequent severe inflammatory responses.

When encountering septic shock and multiorgan failure in a patient, the possibility of aortic dissection should be considered even in the absence of relevant risk factors or suggestive medical history. In conclusion, our case can serve as a reminder that aortic dissection should be considered as a potential cause of septic shock.

## Figures and Tables

**Table 1:** Evidence supporting the diagnosis of septic shock.

Laboratory examination	First day of admission	Second day of admission
WBC (/L)	24.5 × 10^9^	18.5 × 10^9^
CRP (mg/L)	42.4	12
NEUR (%)	90.40	84.8
PCT (*μ*g/L)	60.760	100
ALT (U/L)	517.0	860
AST (U/L)	717.0	1649
Cr (*μ*mol/L)	264	297
BUN (mmol/L)	7.2	7.6
BNP (pmol/L)	970.2	2717
Lactic acid (mmol/L)	15.0	12.3
BEECF (mmol/L)	−20.6	−9.7
PH	7.19	7.35
CO2 (mmHg)	21	23

## References

[1] Hagan P. G., Nienaber C. A., Isselbacher E. M. (2000). The international registry of acute aortic dissection (IRAD): new insights into an old disease. *Journal of the American Medical Association*.

[2] Tupchong K., Koyfman A., Foran M. (2015). Sepsis, severe sepsis, and septic shock: A review of the literature. *African Journal of Emergency Medicine*.

[3] Hicks P., Cooper D. J. (2008). The Surviving Sepsis Campaign: International guidelines for management of severe sepsis and septic shock: 2008. *Critical care and resuscitation : journal of the Australasian Academy of Critical Care Medicine*.

[4] Nguyen H. B., Rivers E. P., Abrahamian F. M. (2006). Severe sepsis and septic shock: review of the literature and emergency department management guidelines.. *Annals of emergency medicine.*.

[5] Spittell P. C., Spittell J. A., Joyce J. W. (1993). Clinical features and differential diagnosis of aortic dissection: Experience with 236 cases (1980 through 1990). *Mayo Clinic Proceedings*.

[6] Luo F., Zhou X.-L., Li J.-J., Hui R.-T. (2009). Inflammatory response is associated with aortic dissection. *Ageing Research Reviews*.

[7] Woodcock N. P., Sudheer V., El-Barghouti N., Perry E. P., Macfie J. (2000). Bacterial translocation in patients undergoing abdominal aortic aneurysm repair. *British Journal of Surgery*.

[8] Shen L., Su L., Turner J. R. (2009). Mechanisms and functional implications of intestinal barrier defects. *Digestive Diseases*.

[9] Hung T. V., Suzuki T. (2016). Dietary Fermentable Fiber Reduces Intestinal Barrier Defects and Inflammation in Colitic Mice. *Journal of Nutrition*.

[10] Gu J., Hu J., Qian H. (2016). Intestinal Barrier Dysfunction: A Novel Therapeutic Target for Inflammatory Response in Acute Stanford Type A Aortic Dissection. *Journal of Cardiovascular Pharmacology and Therapeutics*.

[11] Tani T., Hanasawa K., Endo Y. (1997). Bacterial translocation as a cause of septic shock in humans: A report of two cases. *Surgery Today*.

